# Habitat Preferences at the Leading Edge of a Marine Bioinvasion

**DOI:** 10.1002/ece3.72475

**Published:** 2025-11-12

**Authors:** Alice Hedensjö, Åsa Strand, Ane T. Laugen

**Affiliations:** ^1^ Department of Environmental Intelligence IVL Swedish Environmental Research Institute Fiskebäckskil Sweden; ^2^ Department of Natural Sciences, Centre for Coastal Research University of Agder Kristiansand Norway

**Keywords:** geographical range limit, habitat suitability modeling, invasive species, niche shift, Pacific oyster, urban marine habitat

## Abstract

To enable the early detection and eradication of invasive species, it is crucial to predict habitats with an elevated risk of invasion. Despite the fact that invaders may display initial habitat preferences and niche shifts during range expansion, studies identifying habitat associations at invasion fronts are lacking, especially those considering abundance distributions. We developed a targeted Habitat Suitability Modeling approach to predict invasion risk, focusing on the Pacific oyster (*Magallana gigas*) invasion front on the Swedish southwest coast. We show that marinas act as environmental “hotspots” for pioneering non‐native populations across broad spatial scales. The abundance observed in marinas (10.4 ind. m^−2^) was higher than that in both piers (3.3 ind. m^−2^) and natural rocky habitats (2.8 ind. m^−2^). In terms of invasion risk, marinas were predicted to promote seven times higher oyster abundance and 20 times higher oyster biomass per unit area than natural rocky habitats. While the availability of stable hard substrate influenced presence, shelter from waves influenced abundance, demonstrating the ecological distinction between species occurrence and abundance distributions with important management implications. Moreover, supporting recent genetic findings, our model reveals an unexpected low‐salinity tolerance at the invasion front, suggesting that range expansion may rather be limited by a lack of stable substrate. Our study provides novel insights into the dynamics of marine bioinvasions at leading range edges and offers a practical tool to inform early detection and proactive management of marine invasions, especially in commonly invaded anthropogenically structured habitats such as marinas.

## Graphical abstract

We developed a targeted Habitat Suitability Modeling approach to predict invasion risk during species range expansion, focusing on the Pacific oyster (*Magallana gigas*) invasion front on the Swedish southwest coast. Our findings highlight marinas as key invasion hotspots, supporting markedly higher oyster abundance and biomass than natural habitats. The model also identifies environmental drivers of occurrence and abundance and reveals an unexpectedly low‐salinity tolerance at the invasion front, offering valuable insights for early detection and management of marine invasions.

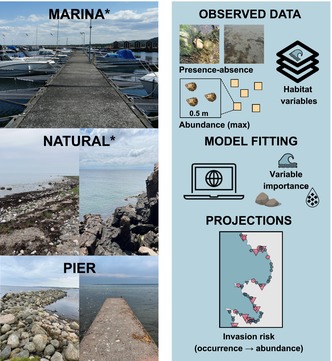

## 1 Introduction

Invasive species are one of the most severe human‐induced threats to global biodiversity (e.g., Mollot et al. 2017). Marine species introductions are mostly driven by shipping and aquaculture (Tricarico et al. 2016). Once introduced, invaders can disperse naturally, further extending their range (Wood et al. 2021). While early interventions are the most effective long‐term strategy to reduce overall impact (Geburzi and McCarthy 2018), invasive species management is often reactive rather than proactive due to a priority of core invaded areas (Ahmed et al. 2022).

To enable cost‐efficient monitoring, early detection, and eradication of invasive species, it is crucial to identify and predict habitats at risk of invasion. Habitat suitability modeling (HSM, also known as species distribution modeling) has become a popular tool to spatiotemporally predict species invasions (Srivastava et al. 2019). HSMs estimate a species' realized niche by statistically linking field observations to environmental conditions (Guisan et al. 2017). Applying HSM to biological invasions, however, is challenging because the species distribution changes dynamically over the course of invasion. Only when fully established does the invader fulfill the environmental equilibrium assumption underlying HSM, that is, it occupies all available suitable habitats (Gallien et al. 2012). During range expansion, however, species may display an initial preference for certain habitat types (Núñez‐Tobajas et al. 2024). Environmental tolerances may also differ at the range edge due to ecological or evolutionary reasons (i.e., niche shifts; Battini et al. 2019). As a result, HSM calibrated on established populations may fail to predict invasion hotspots at the expansion front (Gallien et al. 2012). Yet, there is a deficiency of studies focusing on species‐habitat associations at invasion fronts, particularly those incorporating species abundance. This in turn hinders the implementation of region‐specific management strategies and early detection efforts.

The Pacific oyster (*Magallana gigas*) is one of the most widespread marine invaders globally (Padilla 2010). Introduced for aquaculture purposes in the 1960s, feral populations have spread intensively along European coastlines in recent years (McAfee and Connell 2021). Currently, as a result of the widespread use of triploid oysters in production, natural dispersal of wild populations is likely the main driver of Pacific oyster expansion in northern Europe, as supported by genetic studies and oceanographic trajectory modeling (Faust et al. 2017; Kinnby et al. 2025; Laugen et al. 2015; but see d'Auriac et al. 2017). While Pacific oysters are generally considered “invasive” in Europe, their ecological and economic impacts are context‐dependent and vary depending on habitat type and population density (Green and Crowe 2014).

Traditionally, shallow depths (< 3 m), temperatures above 20°C and salinities above 20 psu are considered important conditions for the establishment of the oyster in new temperate areas (Wrange et al. 2010). However, recent studies suggest that niche shifts have enabled colonization of colder and less saline habitats than in fully established areas (Pack et al. 2022). For example, Kinnby et al. (2025) recently showed that Pacific oysters have locally adapted to low‐salinity conditions and can reproduce at salinities as low as 8 psu at the European invasion front in Sweden. This finding contradicts the hypothesis that a decline in salinity limits the southward spread (Wrange et al. 2010) and suggests a possible Pacific oyster range expansion into the Baltic Sea.

The Swedish southwest coast shows, in addition to a strong southward salinity gradient (from about 25 to 10 psu), a homogeneous coastal morphology characterized by extensive sandy beaches exposed to wave action. Wave exposure can affect the abundance of introduced bivalves (Branch et al. 2008), and Pacific oysters generally tend to be more abundant in sheltered than in wave‐exposed areas (Greeve 2025), such as in marinas (Teschke et al. 2020) or on sheltered sedimentary shores (Reise et al. 2017). Initial Pacific oyster populations in Sweden, restricted to the northwest Swedish west coast (Wrange et al. 2010), predominantly originated from wild populations in Denmark (Faust et al. 2017), as suggested by genetic findings (Faust et al. 2017) and oceanic trajectory modeling (Laugen et al. 2015). In the past two decades the oyster has shifted its southern range limit in Sweden about 1.5 latitudes south, from the Varberg area (Wrange et al. 2010) to Malmö, through dispersal of these northern populations (Kinnby et al. 2025). In this region, referred to as the invasion front, qualitative field observations suggest that the oyster often cements to hard substrates, such as those found in marinas or on piers, but its habitat preferences have not previously been quantified.

Artificial structures provide novel substrates in otherwise space‐limited coastal ecosystems and are often the first to be colonized by invasive species (e.g., Airoldi et al. 2015; Dafforn 2017; Firth et al. 2021; Glasby et al. 2007). Marinas offer a unique combination of both available space and protection from wave action, which rarely occurs along sheltered rocky shores (Lubchenco and Menge 1978) or in more wave‐exposed artificial habitats such as piers or wind farms. Accordingly, local‐scale temporal abundance observations in Helgoland suggest that marinas favor an initial establishment of Pacific oyster populations (Teschke et al. 2020). Whether this invasion dynamic is consistent over broad spatial scales, however, remains unexplored.

This study therefore aimed to quantify habitat preferences at the Pacific oyster invasion front, focusing on the Swedish southwest coast, to predict invasion risk during range expansion. Specifically, we identified habitat types most at risk of invasion and assessed key environmental factors linked to population establishment. By conducting a targeted study design across artificial and natural habitat types and applying HSM, we predicted invasion risk in terms of both occurrence and abundance and evaluated the influence of environmental variables using statistical and machine‐learning approaches.

## 2 Methods

### 2.1 Field Surveys and Analyses of Observed Habitat‐Type Association

**FIGURE 1:**
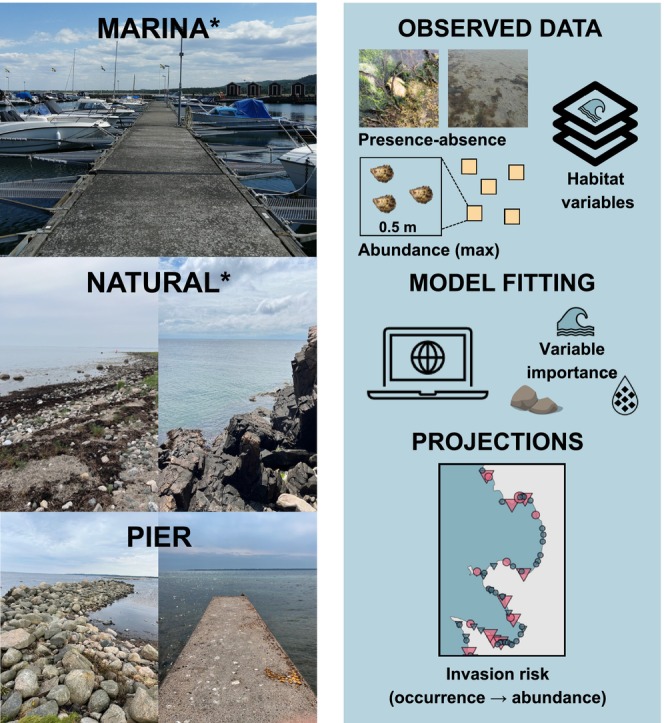
Examples of sites from the three habitat categories surveyed (left) and habitat suitability modeling workflow to predict invasion risk of *Magallana gigas* during range expansion (right). Observed data includes square surveys and environmental GIS data. Habitat categories included in model projections are denoted with an asterisk (see text for details).

**FIGURE 2:**
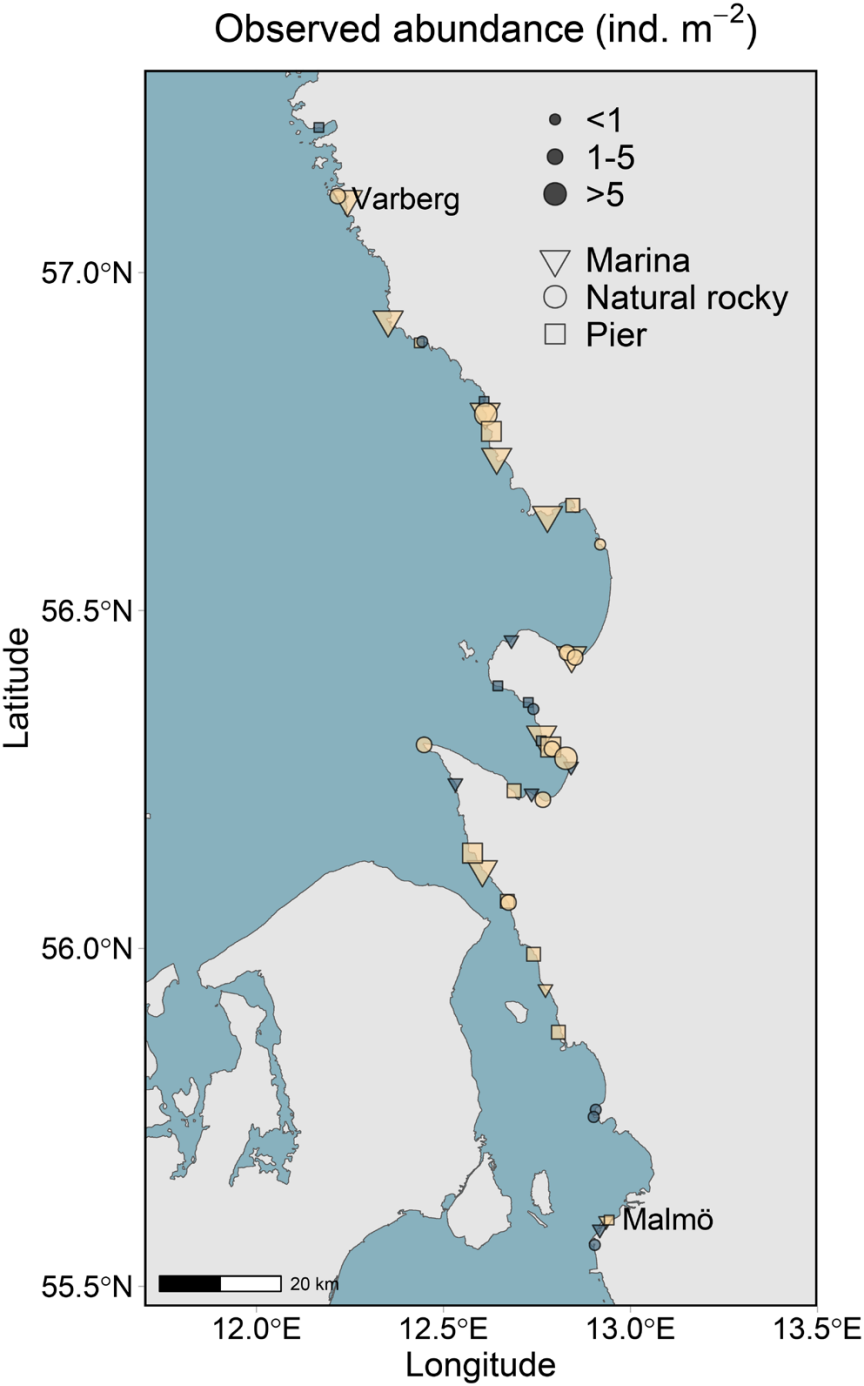
Surveyed sites and observed *Magallana gigas* presence (orange/light) and absence (blue/dark) in marinas, piers and natural rocky habitats on the Swedish southwest coast. The size of the shapes represents observed abundances.

We conducted field surveys of Pacific oysters from June to August 2023 across 45 randomly selected sites divided into three habitat types—marinas, piers, and natural rocky habitat—between Varberg (57.2°N, 12.2°E) and Malmö (55.5°N, 12.9°E, Figures 1 and 2). We selected these habitat categories to capture key environmental variability and to evaluate a potential preference for urban habitats along the invasion front, as indicated by qualitative observations. Natural rocky habitats were defined as areas with the presence of stable hard substrates (exceeding 5 cm in diameter). To avoid spatial autocorrelation issues, sites were separated by at least 100 m (Guisan et al. 2017).

We visually inspected each site for Pacific oysters (hereby oysters) from the high‐water line to 1 m depth. This depth range was selected to maximize the likelihood of encountering the species and the highest abundance area, as indicated by HSMs from other areas in Sweden (e.g., Bergström et al. 2021). In addition, the focus on shallow depths aimed to inform early management efforts, which in practice are often restricted to clearing methods that do not require diving, such as wading or scraping off docks.

At sites where oysters were present, sampling was conducted in the visually identified highest‐abundance area. The purpose of this targeted sampling approach was not to estimate average site‐level abundance, but to minimize the false absence rate and to efficiently capture habitat features most susceptible to early oyster invasion under generally low‐density conditions and environmental non‐equilibrium. We surveyed abundance and habitat structure (e.g., relative substrate cover) using five 0.5 × 0.5 m metal quadrats placed within a sampling plot sized according to a visual assessment of the overall substrate inclination (> 50% vertical surface: 10 × 0.5 m, 20%–50%: 10 × 1 m, 0%–20%: 10 × 10 m). Living shell lengths (from shell edge to umbo) were measured for all oysters in the sampling plot if they counted less than 100; otherwise only oyster sizes in the rectangles were measured. To capture small‐scale habitat associations while minimizing sampling bias, we placed the first sampling quadrat to maximize oyster count, while the placement of the remaining four was randomized within the sampling plot. To reduce the risk of detection bias, the sampling plot was thoroughly inspected (including searches beneath vegetation) before placing the first quadrat. If the oysters were absent at a study site, we randomly distributed the five sampling quadrats in the inspected site area to record the habitat structure.

### 2.2 Habitat Suitability Modeling

To model the risk of oyster invasion and population establishment, we employed a two‐part HSM approach in R version 4.3.3 (R Core Team 2024, Figure 1). First, we modeled presence‐absence using an ensemble modeling approach in *biomod2* (Guisan et al. 2017; Thuiller et al. 2024). Second, we modeled abundance using random forest regressions in *caret* (Kuhn 2008). Machine learning has received growing attention as a powerful tool for abundance modeling (e.g., Greeve 2025). Bagging techniques are, however, less prone to overfitting small sample sizes (< 40) than boosting methods (Luan et al. 2020), hence our choice of random forest.

### 2.3 Selection of Environmental Predictors

We fitted occurrence and abundance models with three environmental predictors: percent cover of hard substrate, wave exposure and minimum salinity. These predictors represent key environmental variability both among and within the three selected habitat categories and may influence invasion‐front dynamics. We limited the number of predictors to comply with the 1 predictor:10 presences rule of thumb (Peduzzi et al. 1996).

We obtained substrate data from field surveys while wave exposure and salinity estimates (resolution 25 × 25 m) were extracted from spatial models (Wennberg et al. 2006) and satellite imagery (CMEMS, 2024), respectively. We depth attenuated the wave exposure data (Bekkby et al. 2008) to obtain representative small‐scale exposure conditions. To account for the enclosure of marinas by breakwaters, not accounted for by the exposure model in certain marinas, we substituted marina exposures exceeding 10,000 m^−2^ s^−1^ (i.e., classified as above “very sheltered,” Wennberg et al. 2006) with the average exposure in marinas below this threshold. Prior to model fitting, we inspected the habitat variables for potential correlation structures to avoid multicollinearity issues. As thresholds for variable inclusion, we used Pearson *r* < 0.70 and VIF < 5 (Guisan et al. 2017).

### 2.4 Occurrence Modeling

To ensure accurate representation of oyster‐substrate associations, we fitted the occurrence model with habitat variables only from the first sampling square across sites, as this square consistently represented the type of substrate the oyster was located on whenever it was present at a site. Candidate models for the ensemble models were fitted with four algorithms: General Linear Models, General Additive Models, General Boosting Models and Random Forest. We set the modeling option to “bigboss,” a set of model parameters recently developed to improve model performance (Thuiller et al. 2024). Models were cross‐validated using repeated split sample (Guisan et al. 2017), where the data were randomly split into 80% training data and 20% validation data. The purpose of the training data is to calibrate the models while the validation data is used to evaluate the predictive performance of the trained models on “unseen” (semi‐independent) data. We repeated this cross‐validation process 100 times, allowing for stable internal evaluations of the models. Based on a visual inspection of the evaluation metrics, we included models that were at least “fair” in the final ensemble model, with validation area under the curve (AUC) and true skill statistic (TSS) of at least 0.70 and 0.50, respectively (Araújo et al. 2005; Duan et al. 2014; Nüchel et al. 2018). The final ensemble model was evaluated based on the mean calibration AUC of included models.

### 2.5 Abundance Modeling

To avoid bias in abundance predictions, the abundance model was fitted with site estimates (average across all five replicate squares) of abundance and habitat variables across sites of observed presence. Although machine learning models lack assumptions in the data distribution, we log transformed the abundance data prior to model fitting, as recommended to increase the variance explained by the model (Martín et al. 2021). Random forest regressions were trained with a sixfold cross‐validation approach with 10 repeats (Guisan et al. 2017), yielding five abundance points in each validation set (Hill et al. 2017). We visually confirmed a decline and stabilization in the error rate before 500 trees. We selected the final abundance model based on the smallest validation root mean square error (RMSE). To correct for the tendency among machine learning regression models to overpredict low values and underpredict high values, we applied a regression of observed on estimated values (Belitz and Stackelberg 2021) before back‐transforming log abundances applying Duan's smearing estimate (Duan 1983).

### 2.6 Analyses of Environmental Predictor Influence

To enable inter‐model comparisons, we applied the same methods for analyzing predictor influence in occurrence and abundance models. The predictor importance was calculated as the average 1‐Pearson *r* between the original predictions and those after permuting the habitat variable, averaged over three runs (Thuiller et al. 2024). To explore the continuous effect of environmental predictors, response curves were generated based on the evaluation strip method (Elith et al. 2005). Curves were based on 100 model predictions within the observed interval of each environmental variable, with other variables kept constant at their median (Thuiller et al. 2024). To assess potential interactions among predictors, we generated three‐dimensional partial dependence plots in *pdp* (Greenwell 2017).

### 2.7 Model Projections and Estimation of Possible Population Sizes

We spatially projected the presence‐absence model onto all 54 marinas along the surveyed coast, as well as onto 108 randomly selected natural sites in the 0–1 depth range. The reason for using a sample projection set for natural habitats, compiled using a depth raster (25 × 25 m resolution, Wennberg et al. 2006), was the lack of quantitative substrate data for the area preventing full‐covering projections.

**FIGURE 3:**
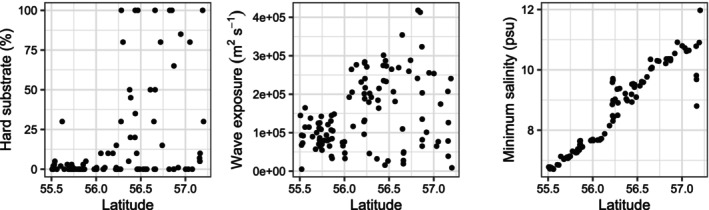
Environmental conditions at natural prediction sites (*n* = 108) plotted against latitude.

For each natural site, we visually estimated the hard substrate cover using satellite images, while salinity and depth‐attenuated exposure values were extracted from raster layers (Figure 3). We did not limit occurrence predictions to rocky sites, as the model was trained to predict in the full range (0%–100%) of hard substrate cover. The total area of natural habitats was estimated using depth raster, and the total area of rocky habitats was estimated based on the prevalence of rocky sites (% hard substrate > 0) in the sample projection set.

In marinas, we assumed a hard substrate coverage of 100% and a constant substrate depth of 0.5 m. This depth limit was used not to overestimate the total habitat area of marinas, which shallower than 1 m mostly consisted of floating docks. The total habitat area of marinas was estimated by multiplying the total perimeter of docks and inner walls, visually assessed using satellite images, by the total area between 0 and 0.5 m depth. As when constructing the models, we substituted exposures in marinas exceeding 10,000 m^−2^ s^−1^ with the average exposure of marinas below this threshold, followed by depth attenuation.

We excluded piers from the model projection as their depth and percentage of hard substrate (often a mixture of hard substrate and other substrate types such as gravel) were infeasible to representatively estimate or assume. While this exclusion may have underestimated the predicted invasion risk, the contribution of piers to parameter estimates was likely negligible, as they were visually estimated to provide orders of magnitude less habitat surface than marinas and natural habitats.

We predicted abundance across areas of predicted occupancy (Greeve 2025). Presence–absence predictions were distinguished using the ensemble model's default cutoff value, optimized to maximize sensitivity and specificity (Thuiller et al. 2024). Since sampling focused on the highest‐abundance area (which may or may not have represented the average abundance of each site), we considered predictions to represent the susceptibility to population establishment (i.e., *possible* abundances) rather than true current abundances.

The *possible* population size ($N_{h}$) in each habitat type was estimated as:

$$N_{h}={\bar{x}}_{h}P_{h}A_{h}$$

where ${\bar{x}}_{h}$ is the mean predicted population size, $P_{h}$ is the predicted prevalence and $A_{h}$ is the areal extent of each habitat type. The *possible* biomass (live wet weight) in each habitat was then estimated using the modeled population size, measured shell lengths and a length‐weight regression based on over 1000 individual Pacific oyster observations (> 0–300 mm) on the Swedish west coast (Å. Strand et al. unpublished data). Parameter uncertainties were estimated with bootstrapped 95% confidence intervals.

## 3 Results

### 3.1 Observed Pacific Oyster Occurrence and Abundance

**FIGURE 4:**
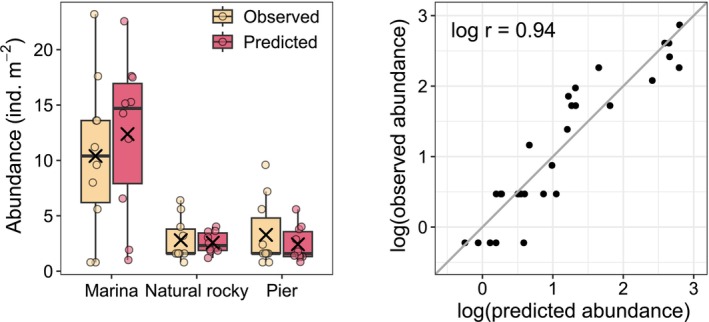
Observed versus predicted *Magallana gigas* abundance. Left: Boxplots of observed and back‐transformed predicted abundances across habitat types (x = mean; line = median). Right: Observed versus predicted log‐abundances where log *r* is the Pearson correlation coefficient. The line is the 1:1 line where observations equal predictions.

We observed Pacific oysters in 30 out of the 45 surveyed sites (66.7%), and presences were distributed across the entire range of the surveyed region (Figure 2). Abundance ranged from 0.8 to 6.4 across natural rocky habitats and to 9.6 ind. m^−2^ across piers. Marinas showed the highest within‐group variability, ranging from 0.8 to 23.2 ind. m^−2^ (Figure 4).

Although oyster prevalence was identical across all habitat types (10 presences in each), an ANOVA on log‐transformed abundances (*F*_2,27_ = 4.6, *p* = 0.02) revealed that marinas supported substantially higher mean abundance (10.4 ind. m^−2^) than both piers (3.3 ind. m^−2^, Tukey post hoc test, *p* = 0.04) and natural rocky habitats (2.8 ind. m^−2^, Tukey post hoc test, *p* = 0.04). In contrast, there was no difference in abundance between piers and natural rocky habitat (Tukey post hoc test, *p* = 0.99). The data complied with parametric assumptions (normality of error and homogeneity of variance), based on graphical assessment prior to analysis.

### 3.2 Environmental Influence on Occurrence and Abundance

**FIGURE 5:**
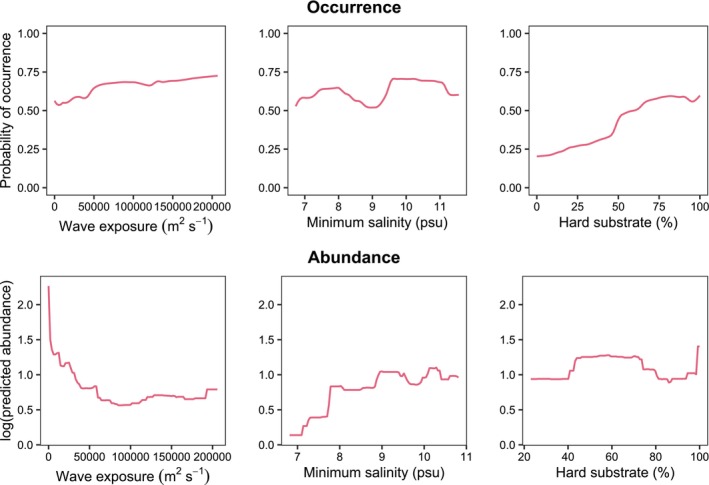
Response of predicted occurrence (top row) or log‐abundance (bottom row) of *Magallana gigas* to observed changes in wave exposure, salinity, and hard substrate cover. The abundance model is calibrated on the observed range of environmental conditions across presence sites.

The presence‐absence model performed excellently (AUC = 0.96), with high sensitivity (proportion of presences correctly predicted = 0.95) and specificity (proportion of absences correctly predicted = 0.93) at a default cutoff (0.55) that maximized both measures. The ensemble consisted of 148 individual models (40% of generated models) which exceeded the validation thresholds (AUC = 0.7, TSS = 0.5). Hard substrate cover was the strongest occurrence predictor (0.67), followed by salinity (0.15) and wave exposure (0.11). Occurrence probability increased with greater hard substrate coverage (Figure 5), aligning with our field observations of oysters cementing exclusively to hard surfaces.

The abundance model also performed well (cross‐validation RMSE = 0.89), with predictions aligning with the observed values (log *r* = 0.94, Figure 4). The mean absolute error between observed and predicted abundances was 1.4 ind. m^−2^, approximately 6% of the observed abundance range. Wave exposure was the most important predictor of abundance (0.47), followed by salinity (0.30). Whereas occurrence probability showed a slightly increasing trend with increasing wave exposure, the highest predicted abundances occurred in the most sheltered sites (Figure 5). Similarly, while occurrence probability showed no trend with salinity, predicted abundance declined sharply below a minimum salinity of 8 psu (Figure 5). Moreover, hard substrate conditions across presence sites (i.e., > 20% cover) had negligible influence on abundance (0.03), although showing two peaks at about 60 and 100% cover (Figure 5).

**FIGURE 6:**
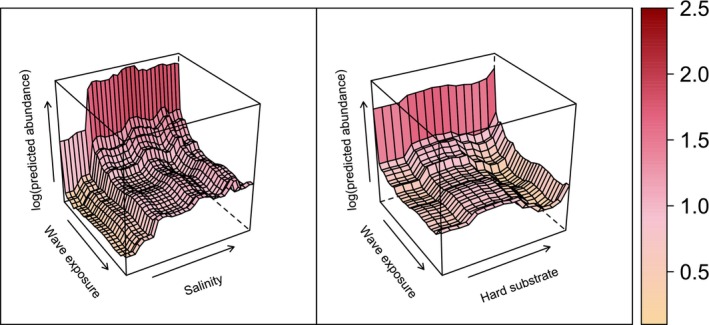
Partial dependence plots showing the joint effect of wave exposure and salinity (left) or hard substrate (right) on abundance predictions. Arrows points towards higher values and darker red colors indicate higher marginal effects on log‐abundance. Wave exposure has the same scaling in both plots.

Joint environmental effects on predicted abundance aligned well with the one‐dimensional response curves (Figures 5 and 6), indicating a lack of strong synergistic or antagonistic interactions within the sampled environmental space.

### 3.3 Spatial Predictions of Invasion Risk

**FIGURE 7:**
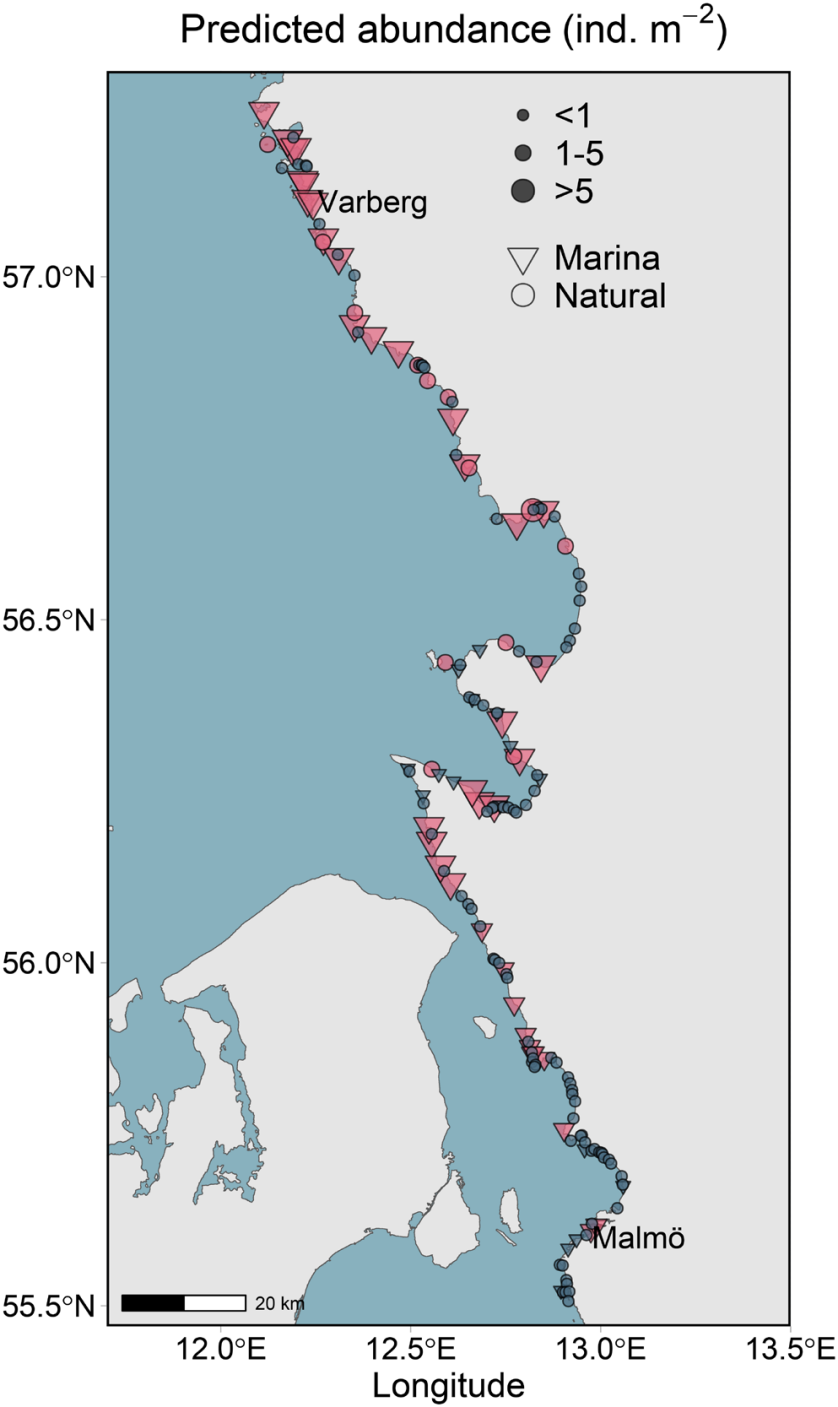
Predicted invasion risk of *Magallana gigas* on the Swedish southwest coast. Predicted presence (pink/light) and absence (blue/dark) for marinas (*N* = 54) and natural habitats (*n* = 108). *Possible* abundance predictions are for sites of predicted presence. Note that no abundance prediction is less than one.

Although prevalence was equal across habitat types, the occurrence model predicted the oyster to be present (i.e., occurrence probability > 0.55) in 70% of marinas, 27% of natural rocky habitats and 12% of all natural habitats (Table 1). The oyster was predicted to be absent in areas lacking hard substrate (Figure 7, Table 1). The total predicted area at risk of invasion was approximately 9 km^2^ (Table 1).

**TABLE 1:** Predicted population parameters of *Magallana gigas* in marinas and shallow (0–1 m) natural habitats on the Swedish southwest coast, between Varberg and Malmö. Rocky habitats are defined by the presence of hard substrate. Bootstrapped 95% confidence intervals for population parameter estimates are in parentheses.

Habitat type	Predicted prevalence (%)	Area of predicted invasion (km^2^)	Predicted *possible* population size (ind. ·10^6^)	Predicted *possible* biomass (t)
Marina	70.4	0.02	0.14 (0.12, 0.17)	16 (13, 18)
Natural rocky	27.1	8.6	26 (21, 31)	1009 (835, 1213)
Natural overall	12.0	8.6	—	—

Abundance predictions ranged from 1.3 to 18.3 ind. m^−2^, a slightly narrower range than observed, despite bias correction for overprediction of low values and underprediction of high values. Sites with predicted high abundance (> 5 ind. m^−2^) were concentrated in the northernmost part of the study area, as well as north and south of the two central headlands (Figure 7). This pattern aligned with field observations, where high‐abundance sites were found exclusively north of 56.1°N (Helsingborg region, Figure 7). South of 56.3°N, the oyster was predicted to be absent from natural habitats (Figure 7), coinciding with a rapid decline in the availability of natural hard substrate, rarely exceeding 10% cover beyond this point (Figure 3).

Marinas were predicted to support three times higher oyster abundance (mean [95% CI] = 8.6 [7.3, 10.4] ind. m^−2^) than natural habitats (mean [95% CI] = 3.0 [2.5, 3.6] ind. m^−2^) across areas of predicted occupancy. Due to the larger habitable area of rocky habitats, however, the predicted *possible* population size and biomass were orders of magnitude greater in natural habitats than in marinas (Table 1). Nonetheless, when standardizing for total available habitat, marinas were predicted to be susceptible to seven times higher oyster abundance and 20 times higher biomass per unit area than natural habitats (Table 1).

**FIGURE 8:**
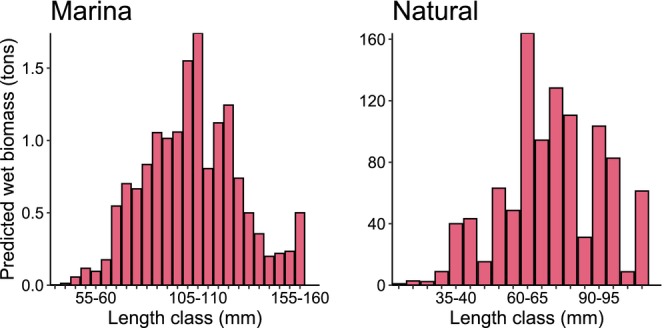
Contribution of *Magallana gigas* length classes to the predicted *possible* biomass in marinas and natural rocky habitats on the Swedish southwest coast.

The high biomass per unit area in marinas was driven by the larger size of oysters compared to those in natural habitats (Figure 8). In marinas, the 105–110 mm size class (about 140 g) contributed most to predicted biomass, whereas the 60–65 mm size class (about 40 g) dominated natural habitats (Figure 8).

## 4 Discussion

In this study, we demonstrate that a combination of targeted field observations and HSM can capture habitat associations at invasion fronts. Our field observations of oysters exclusively cemented to hard substrates and high abundances in marinas closely align with model predictions. These insights have direct implications for early monitoring and targeted management at invasive range edges.

### 4.1 Habitat Preferences and Marina Facilitation

We show that marinas constitute environmental “hotspots” for pioneering Pacific oyster populations. This finding aligns with a growing body of evidence suggesting that artificial marine structures act as invasion foci for sessile non‐native species (e.g., Airoldi et al. 2015; Dafforn 2017; Firth et al. 2021; Glasby et al. 2007). While wave shelter has previously been suggested to facilitate the early establishment and growth of Pacific oyster populations in marinas (Teschke et al. 2020), our findings highlight the potential role of stable hard substrates in facilitating the initial colonization (i.e., occurrence). However, the absence of the species from habitats lacking stable substrates, as predicted here for the invasion front, is not a general phenomenon. In core distributional areas in Europe, the oyster has extensively established in sedimentary habitats, with abundances exceeding 1000 ind. m^−2^ (Reise et al. 2017).

While Pacific oysters are considered generalists, a preference for stable substrates may arise if the hydrodynamic conditions are seldom, in space or time, sufficiently calm for the oysters to establish on sedimentary shores and stabilize the substrate by their own mass. Interestingly, a series of field experiments recently demonstrated that the addition of stable artificial structures enabled Pacific oyster range expansion in an intertidal marsh (Fivash et al. 2021). This “stepping‐stone” function of stable structures (e.g., offshore wind turbines) for sessile species has also been suggested on regional scales (De Mesel et al. 2015; Molen et al. 2018; Wood et al. 2021), and potentially underlies a limited influence of wave exposure relative to hard substrate availability in models of oyster presence (this study, Kochmann et al. 2013).

While wave‐exposed habitats can facilitate the recruitment of opportunistic species by providing bare space for settlement, they are also expected to have a high population turnover due to frequent disturbance (Dayton 1971). Consistent with this, we observed a slight increase in the oyster's occurrence probability but a rapid decline in predicted abundance with increasing wave exposure. On sheltered rocky shores, reduced wave exposure may not translate into population growth due to strong biotic pressures like interspecific competition and predation (Lubchenco and Menge 1978). Perhaps linked to these population trade‐offs, the abundance of Pacific oysters on rocky shores has, corresponding to our observations, generally remained low (< 10–50 ind. m^−2^, Kochmann et al. 2013; Krassoi et al. 2008; Ruesink 2007).

In marinas, however, sessile non‐native species have commonly been reported to be both prevalent and abundant (e.g., Bishop et al. 2015; Connell 2001; Glasby et al. 2007). For example, Teschke et al. (2020) showed on a local scale that marinas promoted a five times higher abundance of Pacific oysters than wave‐exposed artificial habitats after 7 years of colonization, corresponding to our large‐scale abundance observations in marinas versus piers. Moreover, the non‐native kelp *Undaria pinnatifida* has been reported to be about twice as prevalent in marinas than in natural rocky habitats in the UK (Epstein and Smale 2018), aligning with our occurrence predictions. Building upon these observations, we suggest that marinas may act in a two‐stage manner to support the establishment of non‐native species. Extensive access to bare hard substrates (i.e., space) may facilitate the initial colonization (occurrence), while protection from waves may enhance population persistence (abundance).

It is important to note, however, that species‐environment relationships predicted here are, although ecologically plausible, correlative. Although Pacific oysters tend to be more abundant in sheltered than in exposed sites (e.g., Greeve 2025), an alternative explanation for marinas favoring high abundances is the unique habitat characteristics of floating docks. While previous work suggests that floating structures may promote epifaunal abundance due to their constant depth relative to the water surface (Connell 2001), this mechanism is less plausible in Sweden due to minimal tidal range (~0.3 m). Moreover, Teschke et al. (2020) found a higher Pacific oyster abundance inside than outside a marina despite comparing similar fixed artificial structures, suggesting wave exposure as the more influential factor.

Biotic interactions, or the lack thereof, may also contribute to the proliferation of invaders in marinas. Predation seems to have suppressed impact on fouling communities in marinas (Leclerc and Viard 2018), with the position of floating docks and pilings relative to shore as a potential mechanism limiting the colonization of native species (Glasby et al. 2007). In contrast, Firth et al. (2021) showed that, while the colonization of primary invaders, such as Pacific oysters, was facilitated by the availability of bare substrate in artificial environments, native rather than invasive epibionts were favored during secondary colonization in artificial habitats. While complex biotic interactions were beyond the scope of this study, the combination of substrate availability and wave shelter, as evident in our study, appears at least to be a useful indicator of primary invasion risk.

Another important consideration is the potential influence of anthropogenic vectors on population patterns. A possible explanation for marinas emerging as early hotspot habitats for invasive species is an elevated supply of larvae in these habitats due to, for example, the release of ballast water. However, while human‐mediated vectors such as ballast water enable introductions beyond natural dispersal limits, this artificial contribution of larvae is likely negligible once feral populations are established on a regional scale, as long as there are no limits to natural dispersal. Several lines of evidence point towards high Pacific oyster abundances in marinas on the Swedish southwest coast being sustained by favorable environmental conditions rather than an artificially elevated propagule pressure. Firstly, Swedish oysters are genetically similar and predominantly of wild origin (Faust et al. 2017). Secondly, we observed established oysters in marinas at the invasion front indicating low‐salinity adaptation within a single expanding population (Kinnby et al. 2025). Thirdly, commercial aquaculture activities involving the species have never been established in Sweden. Finally, we observed high abundances in non‐industrial harbors unlikely to receive a continuous and large supply of ship‐derived larvae. Thus, in line with local‐scale evidence (e.g., Teschke et al. 2020), our study demonstrates that marinas can function not only as artificial entry points for invasive species, as traditionally emphasized, but also as environmental hotspots that promote invasive population proliferation at range edges under natural dispersal.

### 4.2 Niche Shifts and Local Adaptation

Niche shifts have been increasingly recognized as critical components of invasive species dynamics (Battini et al. 2019). They can occur due to phenotypic plasticity, biotic interactions and rapid evolution (Guisan et al. 2014). In line with recent evidence of local adaptation (Kinnby et al. 2025), we show that Pacific oysters have successfully invaded low‐salinity conditions of the Swedish southwest coast. The predicted abundance did not decline until a minimum salinity of 8 psu, indicating that these conditions can sustain viable oyster populations in the area. Moreover, salinity had a neglectable influence on occurrence, suggesting that sink populations may be maintained in the oyster's southernmost range by annual changes in the prevailing northward currents (Broström et al. 2024). Kinnby et al. (2025) conclude that low‐salinity tolerant Pacific oysters have developed on the Swedish southwest coast through both acclimatization and natural selection, with genetic components almost exclusively accounting for observed differences in fertilization rates at 8 psu at the present invasion front. This adds to a growing number of studies emphasizing the adaptive capacity and phenotypic plasticity of invaders (Allendorf et al. 2022 and references therein).

Given that Pacific oysters have successfully colonized low‐salinity conditions within just two decades, it may only be a matter of time before they expand their Swedish range into the Baltic Sea. Intriguingly, we suggest that the absence of Pacific oysters south of Malmö is due to the lack of hard substrate rather than, or alongside, low‐salinity conditions. This novel hypothesis must be tested experimentally, but is consistent with our findings and first records of the oyster in the Baltic Sea proper, which were found attached to stable structures (Ewers‐Saucedo et al. 2020).

### 4.3 Modeling Considerations and Limitations

Few (if any) studies have explicitly applied habitat suitability models to predict invasion risk, while directly comparing artificial and natural habitats. Most previous HSM applications have focused on predicting potential distributions in natural habitats (e.g., Bergström et al. 2021). Where artificial habitats have been considered, studies have focused on within‐group habitat variability rather than treating artificial systems as integral components of the seascape (e.g., Foster et al. 2016), or have included the proximity to marinas as a covariate to predict natural distributions (e.g., Epstein and Smale 2018). Like our study, Airoldi et al. (2015) analyzed regional occurrence and abundance patterns of non‐native species in artificial (sheltered and exposed) and natural habitats, but unlike our study, they did not employ HSM or generate spatial predictions. By explicitly incorporating both artificial and natural habitats into a unified HSM framework, our study advances these approaches by both quantifying and predicting habitat‐specific invasion risk.

Our models, despite showing a high predictive power, face three main limitations. The first is the small amount of calibration data. HSM accuracy is expected to decline below 30 species presence observations (i.e., our sample size) and generally requires at least 50 presences to reduce sensitivity to sample size effects (Guisan et al. 2017). The second is marina assumptions and incomplete GIS data. Model assumptions made for marinas, such as minimum habitat variability aside from a regional salinity gradient (but see Foster et al. 2016), may have reduced the precision of within‐group predictions. Moreover, a lack of quantitative substrate data restricted predictions in natural habitats to less than 1% of the area, increasing uncertainty in parameter estimates and limiting the spatial coverage of model projections. The third is a lack of a long‐term temporal aspect. Our models do not distinguish between unsuitable and unpreferred (yet suitable) habitats, likely underestimating a future realized niche at the present invasion front. For present‐day monitoring purposes, projections based on core‐range models could therefore be useful, e.g., for mapping sedimentary shores suitable for future establishment. Despite these limitations, the strong agreement between observed and predicted distribution patterns, combined with evident environmental trends, supports the robustness of our findings, and suggests that key conclusions hold.

When applying HSM to invasion fronts, we recommend employing a stratified sampling approach that includes marinas and associated environmental covariates to avoid underestimating the invasion risk of non‐native species. Environmental predictors that show regional rather than habitat‐type‐specific variability should, however, be carefully selected based on each specific case. For example, although not considered here, temperature may be an appropriate predictor to include in HSMs for areas in which temperature is hypothesized to limit range expansion, such as the Pacific oyster's northern range limit in Europe.

### 4.4 Management Implications

The ecological distinction between species occurrence and abundance is critical to guide the early detection (occurrence) and proactive management (abundance and biomass) for invasive species, yet rarely accounted for. Most HSMs consider only presence‐absence distributions and lack abundance predictions due to the scarcity of abundance data (Hao et al. 2019) or to poor‐fit abundance models. Yet, spatiotemporal predictions of abundance and biomass are crucial to guide eradication efforts to limit spread and to quantify the ecological impact of species that can alter and create habitats, like the Pacific oyster (Green and Crowe 2014).

To prevent further range expansion of Pacific oysters, particularly into the Baltic Sea, it is crucial to avoid artificial addition of hard substrate in coastal areas. Hard substrate availability appears to be a key limiting factor in the species' southern range, and its deliberate or incidental introduction could further dispersal into low‐salinity areas. At and beyond the current invasion front, targeted monitoring, and removal of oysters from habitats with high invasion risk, especially sheltered and hard‐substrate habitats like marinas, will be essential to limit local population growth and reduce propagule pressure on surrounding areas. The ecological consequences of the Pacific oyster invading the Baltic Sea remain unknown but could be severe, given the region's already high anthropogenic pressure and low biodiversity (Reusch et al. 2018). Implementing preventive measures to limit further spread is therefore critical. Together, the results and recommendations presented here provide a transferable framework for early detection and targeted control of marine invaders in urbanized coastal environments.

Targeted clearing of Pacific oysters in marinas may help reduce larval “spillover” to adjacent natural habitats, especially if marinas act as local source populations. Marinas have been shown to source nearby natural rocky habitats with invasive kelp (Epstein and Smale 2018), pointing to the possibility that this also occurs for Pacific oysters. Supporting this, Teschke et al. (2020) showed that the temporal increase in oyster abundance in wave‐exposed artificial habitats lagged that occurring in nearby marinas. Moreover, given the large oyster sizes and numbers in marinas, larval production is likely high (Moore et al. 2016). Despite limited research, further spread into natural rocky habitats is concerning, partly due to findings suggesting that it may initiate a cascade of secondary invasion (Firth et al. 2021). On the other hand, marinas may promote larval retention due to water residence times likely often exceeding the 3–4 weeks oyster larvae spend as plankton before settling (Quayle 1988). Depending on local hydrodynamic conditions, this retention could reduce the number of larvae exported to surrounding areas, while still sustaining high local population densities. To evaluate the effectiveness of oyster removal to prevent spread, it is therefore critical to assess the extent to which marinas act as larval sources for adjacent habitats.

An estimate of the current Pacific oyster population size on the Swedish southwest coast is still lacking, as the purpose of this study was to predict invasion risk rather than current population status. In other words, our findings highlight the importance of active management to prevent the population size from reaching the predicted level of about 60 million individuals and 1000 tons biomass of Pacific oysters. These values represent plausible upper estimates based on model assumptions and should be interpreted as first‐order approximations rather than precise measurements. Our study only considered oysters shallower than 1 m, assuming this zone covers the densest part of the population and thereby represents habitat conditions with the highest invasion risk. While this depth interval is likely the most effective and practically feasible target for early management interventions, Pacific oysters have occasionally been observed at the bottom of marinas (> 3 m) in the study area. This suggests that larval spillover or adult transport from shallower depths may occur within the marina itself and highlights a need to include a larger depth interval for comparative mapping of the oyster distribution in artificial and natural habitats.

While our findings are specific to the Swedish southwest coast, the approach and insights are transferable to other coastal regions facing emerging bioinvasions. Targeted monitoring at invasion fronts, especially in anthropogenically structured habitats like marinas, combined with range‐edge HSMs and abundance modeling, can help identify population hotspots early and inform scalable, evidence‐based management strategies. Broader application of this framework may improve invasive species forecasting and response efforts globally.

## Author Contributions

**Alice Hedensjö:** conceptualization (equal), data curation (lead), formal analysis (lead), investigation (lead), methodology (lead), visualization (lead), writing – original draft (lead), writing – review and editing (equal). **Åsa Strand:** conceptualization (equal), formal analysis (supporting), funding acquisition (supporting), investigation (supporting), methodology (supporting), supervision (equal), visualization (supporting), writing – original draft (supporting), writing – review and editing (equal). **Ane T. Laugen:** conceptualization (equal), formal analysis (supporting), funding acquisition (lead), investigation (supporting), methodology (supporting), resources (lead), supervision (equal), visualization (supporting), writing – original draft (supporting), writing – review and editing (equal).

## Conflicts of Interest

The authors declare no conflicts of interest.

## Data Availability

Data and code used to generate the models and predictions are available in the Open Science Framework repository: https://osf.io/7v84r/?view_only=d9c09262919b465b970757b1b9fdac65.
